# Chronic idiopathic hyperphosphatasia with unusual dental findings - A case report

**DOI:** 10.4317/jced.50878

**Published:** 2012-12-01

**Authors:** Cheriya K. Sreejan, Nair Gopakumar, Gogineni Subhas Babu

**Affiliations:** 1 M.D.S, Assistant professor. Department of Oral Medicine and Radiology, Malabar Dental College, Manoor- Chekanoor road, Mudur .P.O. Kerala, India; 2 M.D.S, Principal, Mahe Dental College, Mahe, India; 3 M.D.S, Professor and Head of department. Department of Oral Medicine and Radiology. A.B. Shetty Memorial Institute of Dental Sciences, Deralakatte, Mangalore, Karnataka. India

## Abstract

Chronic idiopathic hyperphosphatasia(CIH) or juvenile Paget disease is believed to be a distinct disease characterized by an increase in the serum alkaline phosphatase, cortical thickening and bowing of the long bones, especially the femora. It is a rare autosomal recessive bone disorder, with excessive bone resorption and bone formation. Skeletal malformations in the legs may cause problems in walking and may eventually result in short stature. The radiographic appearances include widening of the diaphyses, vertebral osteoporosis, acetabular protrusion, and thickening of the skull vault. Intensive bisphosphonate treatment prevented the development of deformity and disability but there is no published data on long-term efficacy. Bisphosphonate therapy showed suppression of bone turnover, doubling of trabecular thickness with no mineralization defect, and no osteopetrosis. We report a female of 21 years, a case of chronic idiopathic hyperphosphatasia congenital form, with a history of fracture, short stature and malformed teeth. She had a waddling gait, bone deformities, kyphoscoliosis and curvature of her limbs.

** Key words:**Hyperphosphatasia, autosomal recessive, alkaline phosphatase, short stature, cortical thickening, enamel hypoplasia.

## Introduction

Idiopathic hyperphosphatasia is a rare high bone turnover congenital bone disease in which affected children are normal at birth but develop progressive long bone deformities, fractures, vertebral collapse, skull enlargement, and deafness. There is, however, considerable phenotypic variation from presentation in infancy with severe progressive deformity through to presentation in late childhood with minimal deformity (1). Most cases appear to arise from inactivating mutations in the gene encoding osteoprotegerin, a product of osteoblasts that is critically involved in osteoclastogenesis. Treatment with inhibitors of bone resorption (calcitonin or bisphosphonates) showed remarkable clinical and radiographic improvement with normalization of bone markers of osteoblastic and osteoclastic activity (2). We report a female of 21 years, a case of chronic idiopathic hyperphosphatasia congenital form, with a history of fracture, short stature and malformed teeth. She had a waddling gait, bone deformities, kyphoscoliosis and curvature of her limbs.

## Case Report

A 21 year old female patient reported with a complaint of malformed teeth since eruption of the teeth. The patient had noticed the malformation since the time of the eruption of the teeth with no history of sensitivity or pain reported. The patient’s deciduous dentition were also reported to be yellowish but of a milder nature. Past medical history revealed that she had history of convulsions since the age of 6 months and was treated with anticonvulsants (Tab Diamox 250 mg thrice daily) till the age of five years. The patient had a history of delayed milestones and was hospitalized at the age of six years with a complaint of progressive deformity of both legs. Reports showed she had high serum levels of phosphorus (140 mg/dl) and alkaline phosphatase (1149 U/L) and a diagnosis of idiopathic hperphosphatasia was given. She had a history of fracture of her left thigh at the age of 15 years following a minor fall and serum alkaline phosphatase assay was carried out which was then high (346 U/L), but serum phosphorus level was normal. Her parents, brothers and a sister were of normal stature and in good health. There is no family history of consanguinity, skeletal abnormality or dwarfism. The drinking water at her place of residence is not fluorinated.

**Figure 1:**
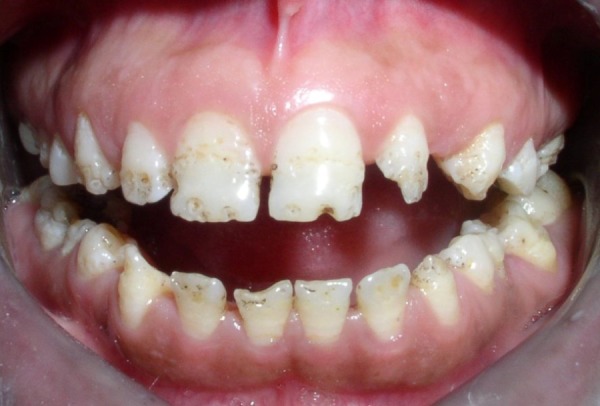
Teeth showing generalized enamel pitting and malformed crown.

On general physical examination the patient was of short stature with short limbs and a normal size trunk. There was an obvious lateral bowing of the legs with slight muscle wasting and no noticeable limitation of movements of both hips. Vital signs and cranial nerve assessment was normal. On head and neck examination the head appeared rather large, but there was no prognathism, exophthalmos or facial paralysis. Intra oral hard tissue examination revealed microdontia with generalized enamel pitting, yellowish discoloration and open contacts between the teeth. Crowns appeared to be malformed (Fig. 1). Full mouth intraoral periapical radiographic survey showed a generalized blunting of root apex with generalized decreased density and reduced thickness of the enamel. Pulp chambers and canals appeared normal.

**Figure 2:**
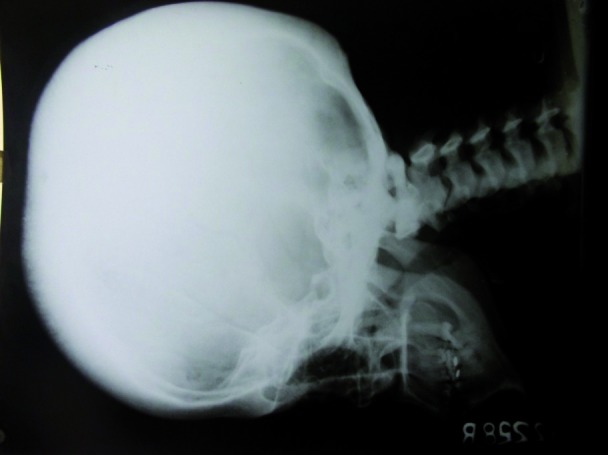
Skull radiograph showed thick and sclerotic skull vault with hypoplastic frontal sinus with mid face hypoplasia.

**Figure 3:**
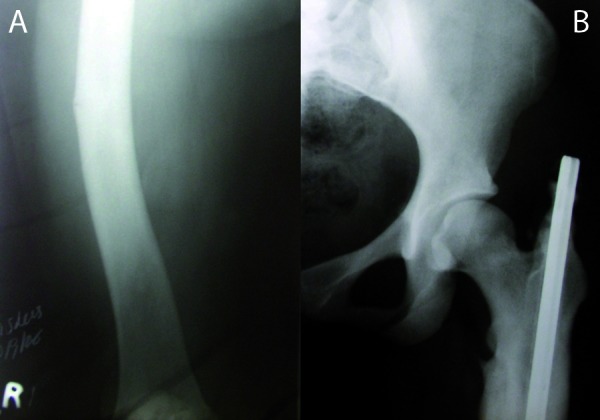
A. Long bone radiographs showed increased density of pelvic bone with a previous fracture site of left femur. B. Right femur showed lateral bowing with a fracture along the lateral aspect at the mid region.

Skull radiograph showed thick and sclerotic skull vault with widened diplo’ and lateral cephalogram showed a hypoplastic frontal sinus with mid face hypoplasia (Fig. 2). Long bone radiographs showed increased density of pelvic bone with a previous fracture site corresponding to the subtrochanteric region of left femur and right femur showed lateral bowing and cortical thickening with a fracture along the lateral aspect at the mid region (Fig. 3). There was bilateral coxa valga deformity.

Biochemical analysis showed an elevated level of serum alkaline phosphatase (192 U/L). The 24-hour urine analysis for calcium, phosphorus and serum phosphorus showed normal values.

The patient was advised to undergo restorations and prosthetic rehabilitation of the malformed teeth.

## Discussion

Hyperphosphatasia refers to disorders that feature elevated serum alkaline phosphatase activity (hyperphospha-tasemia) usually from excesses of the bone isoform of alkaline phosphatase. Rubin (3) in 1964, classified the condition as a diaphyseal dysplasia and distinguished congenita and tarda forms of the disorder. No evidence for chromosomal defect and is probably autosomal recessive. Cortical thickening of the diaphysis of the long bones is a universal feature of the previously reported cases of chronic idiopathic hyperphosphatasia in both children and adults. It is bilateral and symmetric. Roentgenographic changes are similar to those reported for certain cases of osteogenesis imperfecta. Elevated serum alkaline phosphatase has been reported in certain cases of osteogenesis imperfecta although not at the levels found in our patient. Significant findings in congenital hyperphosphatasia included nearly normal at birth with progressive deformity beginning after weeks or months, multiple bone injuries caused by minimum trauma, normal intelligence and a negative family history. On physical examination affected persons are dwarf with large head, saddle nose, blue sclerae, early loss of deciduous teeth, high-frequency hearing loss, warm skin, pigeon breast, bowed extremities with compensatory joint contractures, tenderness or flexibility of bones or both and other organ systems will be normal. Heavy excretion of hydroxyproline peptide in urine is usually found with normal calcium excretion. Teeth showed resorption of dentin by osteoclasts and replacement of pulp by osteoid.

Growth at the epiphyses, epiphyseal plates and metaphyses is normal, but there is overcylinderization of the diaphysis with cortical thickening as in Englemann’s disease. In the latter disorder, however, cortical thickening is more eccentric with fusiform expansion of the diaphysis of the long bones. Roentgenography shows progressive generalized conversion to coarsely trabeculated bone with scoliosis, biconcave vertebral bodies, coxa vara, protusio acetabulae and diaphyseal bowing with relatively normal epiphyses. Subperiosteal layers of new bone on the concave surfaces and multiple diaphyseal transverse dense lines probably representing infractions are also seen. Thickening of the skull vault and base is associated with obliteration of the frontal sinuses in Pyle’s disease (craniometaphyseal dysplasia) but in chronic idiopathic hyperphosphatasia the frontal sinuses are not obliterated and the pituitary fossa is also normal. The basal skull foramina may be obliterated and cause facial paralysis, deafness and optic atrophy. The roentgenologic appearances of the skull vault differ from that seen in Paget’s disease. Fluorosis and myelosclerosis will show other features on skeletal survey to distinguish them from idiopathic hyperphosphatasia. The association of mental retardation and persistent hyperphosphatasia has been described in rare instances, which is in particular characterized by a recognizable facial gestalt and brachytelephalangy (4-9).

In the present patient there were no clinical or roentgenologic features to suggest ankylosing spondylitis, fluorosis or alkaptonuria. Progressive bowing of the femora from childhood, progressive diaphyseal thickening and elevation of the serum alkaline phosphatase, all features which have been described in the congenita form of idiopathic hyperphosphatasia were present in this patient.

The prognosis for survival does not seem unduly grave. They are quite healthy except for their bones. On the other hand, if effective treatment is not provided, the prognosis for a useful life is poor. Normal intelligence notwithstanding, sharply restricted mobility and constant pain will limit physical and mental training. Therapy, to be effective, should slow down both the rate of synthesis and the rate of bone replacement in order to permit adequate remodeling, which would add strength to the presently weak skeleton. A number of empiric medical treatments, including prednisone, acetylsalicylic acid, vitamin D, and dilantin, have been tried on these children because of the reported effect of these drugs on some aspect of bone or collagen metabolism (5). No benefit or obvious harm was produced. Treatment with cyclical intravenous pamidronate induced remarkable clinical and radiographic improvement with normalization of bone markers of osteoblastic and osteoclastic activity, including bone alkaline phosphatase, urinary hydroxyproline, and urinary CrossLaps (10). Intensive bisphosphonate treatment showed suppression of bone turnover and a doubling of trabecular thickness, with no mineralization defect, and no osteopetrosis (11). It may take several more years to evaluate completely the effectiveness of this treatment, even if we are fortunate enough to be able to follow these patients.

## References

[1] Chong B, Hegde M, Fawkner M, Simonet S, Cassinelli H, Coker M (2003). Idiopathic hyperphosphatasia and TNFRSF11B mutations: relationships between phenotype and genotype. J Bone Miner Res.

[2] Cundy T (2002). Idiopathic hyperphosphatasia. Semin Musculoskelet Radiol.

[3] Rubin P (1964). Dynamic Classification of Bone Dysplasias. J Med Educ.

[4] McNulty JG, Pim P (1972). Hyperphosphatasia Report of a case with a 30 year follow up. Am J Roentgenol Radium Ther Nucl Med.

[5] Eyring EJ, Eisenberg E (1968). Congenital hyperphosphatasia: A clinical, pathological, and biochemical study of two cases. J Bone Joint Surg Am.

[6] Olsen CB, Tangchaitrong K, Chippendale I, Graham HK, Dahl HM, Stockigt JR (1999). Tooth root resorption associated with a familial bone dysplasia affecting mother and daughter. Pediatr Dent.

[7] Stemmermann GN (1966). An histologic and histochemical study of familial osteoectasia. (Chronic idiopathic hyperphosphatasia). Am J Pathol.

[8] Thompson MD, Nezarati MM, Gillessen-Kaesbach G, Meinecke P, Mendoza-Londono R, Mornet E (2010). Hyperphosphatasia with seizures, neurologic deficit, and characteristic facial features: Five new patients with Mabry syndrome. Am J Med Genet A.

[9] Horn D, Schottmann G, Meinecke P (2010). Hyperphosphatasia with mental retardation, brachytelephalangy, and a distinct facial gestalt: Delineation of a recognizable syndrome. Eur J Med Genet.

[10] Tau C, Mautalen C, Casco C, Alvarez V, Rubinstein M (2004). Chronic idiopathic hyperphosphatasia: normalization of bone turnover with cyclical intravenous pamidronate therapy. Bone.

[11] Cundy T, Wheadon L, King A (2004). Treatment of idiopathic hyperphosphatasia with intensive bisphosphonate therapy. J Bone Miner Res.

